# The bubble theory: exploring the transition from first replicators to cells and viruses in a landscape-based scenario

**DOI:** 10.1007/s12064-024-00417-4

**Published:** 2024-05-09

**Authors:** Radoslaw W. Piast

**Affiliations:** Chemistry Department, Warsaw University, Pasteura 1, Warsaw, Poland

**Keywords:** Viruses, Proto-viruses, Proto-cells, RNA world, RNP world, Evolution, Abiogenesis, Origin of life

## Abstract

This study proposes a landscape-based scenario for the origin of viruses and cells, focusing on the adaptability of preexisting replicons from the RNP (ribonucleoprotein) world. The scenario postulates that life emerged in a subterranean “warm little pond” where organic matter accumulated, resulting in a prebiotic soup rich in nucleotides, amino acids, and lipids, which served as nutrients for the first self-replicating entities. Over time, the RNA world, followed by the RNP world, came into existence. Replicators/replicons, along with the nutritious soup from the pond, were washed out into the river and diluted. Lipid bubbles, enclosing organic matter, provided the last suitable environment for replicons to replicate. Two survival strategies emerged under these conditions: cell-like structures that obtained nutrients by merging with new bubbles, and virus-like entities that developed various techniques to transmit themselves to fresh bubbles. The presented hypothesis provides the possibility for the common origin of cells and viruses on rocky worlds hosting liquid water, like Earth.

## Introduction

The origin of life remains a profound mystery, but most hypotheses regarding abiogenesis agree that an autocatalytic cycle involving an information-storing molecule must have emerged at some point, giving rise to the RNA world (Cech 2012). Subsequently, this system gained the ability to produce peptides and proteins, which assumed the role of central catalytic units, marking the era known as the RNP world (Cech 2009; Schimmel 2011). The final stage is believed to involve the encapsulation of this chemical system within a lipid-based membrane, resulting in the formation of a fully functional cell (Segré et al. 2001).

In 2000, the International Committee on Taxonomy of Viruses (ICTV) classified viruses as non-living entities, viewing them solely as infectious agents that replicate inside living cells (Zimmer 2021). However, subsequent discoveries of giant viruses (La Scola et al. 2003) and virophages (La Scola et al. 2008) have raised new questions and uncertainties on this matter. As a result, the nature of viruses, their origin, and their place in the tree of life remain the subject of ongoing debate.

Three main hypotheses have been proposed regarding the origin of viruses:

1. The Progressive Hypothesis (Escape Hypothesis) suggests that viruses originated as selfish genetic elements within living cells, acquiring the ability to transfer from one cell to another (Forterre and Krupovic, 2012).
2. The Regressive Hypothesis (Reduction Hypothesis) posits that viruses were once parasitic cells that lost their cellular integrity and reduced their genomes (Baˆndea 1983).
3. The Virus-First Hypothesis considers parasitic replicators in the RNA world as the earliest living entities, gaining the ability to transcribe RNA into DNA. However, the term “virus” in this context might be misleading since it deviates from the modern understanding of viruses, which implies the necessity of parasitism on cellular entities. Instead, this hypothesis is based on the unique ability of these molecular species to perform reverse transcription, suggesting the existence of this strategy before life on Earth transitioned from an RNA-based to a DNA-based genome (Koonin and Martin 2005; Nasir et al. 2012).

## New scenario

The first initial premise for this proposal is the existence of nucleic acids capable of replicating their information through collaboration with proteins. This early stage of nucleic acids–proteins codependency mirrors the contemporary cycle of transcription, translation, and replication observed in living systems. A sustainable autocatalytic cycle with this setup is known as the RNP world—a stage in the origin of life wherein RNA serves as the primary genetic information storage molecule, while proteins fulfill executive functions (Cech 2012; Piast and Wieczorek 2017). This early cycle allowed information to get preserved and to mutate, providing the primitive life-like system operating on canonical evolutionary processes.

The necessity of proteins in this system, despite known functional autocatalytic cycles with only nucleic acids (Neveu et al. 2013), can be rationalized by proteins’ indispensable role in replication and translation processes across all known life forms. Notably, no naturally occurring living system has been discovered relying solely on nucleic acids for the entirety of genetic information maintenance, including replication, transcription, and translation processes, underscoring the codependency of nucleic acids and proteins from the earliest stages of life (Wolf and Koonin 2007). Furthermore, the reverse transcription process essential for introducing DNA into the RNA world is exclusively catalyzed by proteins in nature (Samanta and Joyce 2017), suggesting the precedence of the RNP world over DNA.

Considering these factors, the RNP world serves as the initial framework for this hypothesis. While the scenario proposed aligns with DNA-based biology as well, the plausibility of the RNP world as a precursor to contemporary molecular biology dogma, its higher energetic efficiency, and the fact that it represents a natural minimal requirement for the following scenario, support this choice.

Initially, in the RNP world, pre-cellular genetic elements were selected solely based on their ability to replicate. Genes in the first replicating entities were scattered throughout the Hadean waters, but the evolutionary advantage of compressing useful replication fragments favored their merger (Lawrence and Roth 1996). Thus, we can envision these entities as selfish genes/operons, storing genetic information on a single nucleic acid molecule, serving as replicators as well as replicons.

The second initial premise is the prebiotic production of amphiphiles, like fatty acids, able to create vessels—liposomes. The probability of such molecule’s production and their role in the origin of life has been extensively studied over the years (Segré et al. 2001; Luisi et al. 1999). Production, release, and even loading of the amphiphilic vesicles coupled with concentration and chemical evolution of their content, could occur in one of many proposed mechanisms like 1) a result of multilamellar matrix interaction with water during hydration–dehydration cycles in hydrothermal pools (Damer and Deamer 2015); 2) in alkaline hydrothermal vents (Jordan et al. 2019); 3) as a result of the inverted micelle aerosol reentering water (Tuck 2002). Since amphiphilic vesicles have the ability to mimic the behavior of living cells by displaying growth, merger, division, and even chemotaxis (Maoz et al. 1996; Hanczyc et al. 2003), it is believed that at some point they were playing a crucial role in the origin of life.

Four billion years ago, the Earth had a freshly formed solid surface. A drop of the temperature allowed water to condense and form rivers, lakes, and seas. Many reservoirs could have had an internal volcanic heat source, much like today’s Grand Prismatic Spring in Yellowstone. One such subterranean lake could have been the Darwinian “warm little pond” (Darwin 1871; Damer 2016), which served as an environment for the first replicating systems due to its ability to concentrate organic matter needed for life’s building blocks production (DeGuzman et al. 2014; Follmann and Brownson 2009; Patel et al. 2015) and convective PCR (Braun et al. 2003). Within the waters of this Eden, fatty acids, able to enclose a small portion of this prebiotic soup within liposomes (Fig. 1.1), were produced as well. This Eden was also the birthplace of a stream that flew, let us say east of Eden, as it made its way toward the Hadean Ocean, merging along the way with other streams and rivers in the tributary. The further it went, the more diluted the nutrients became, eventually reaching the dilution point (Fig. 1.2). Here, the concentration of nutrients is diluted beyond the point where replication can be sustained. Hydrolysis starts to dominate over synthesis, and genetic information perishes. The last stand of the original “dense” prebiotic soup is within, created back in the warm little pond, vesicles. These vesicles could also carry replicons. Liposomes hosting a population of replicons are in this work referred to as “bubbles.” Due to the final space of a bubble, replicons have a chance to endure if they manage to adapt to new survival requirements presented by this environment. They must develop one of two possible strategies to ensure their survival. They either found a way to swap vesicles after depleting the resources of the old one (the virus strategy) or increase the probability of the original bubble to fuse with a fresh one (the cell strategy).

**Fig. 1:**
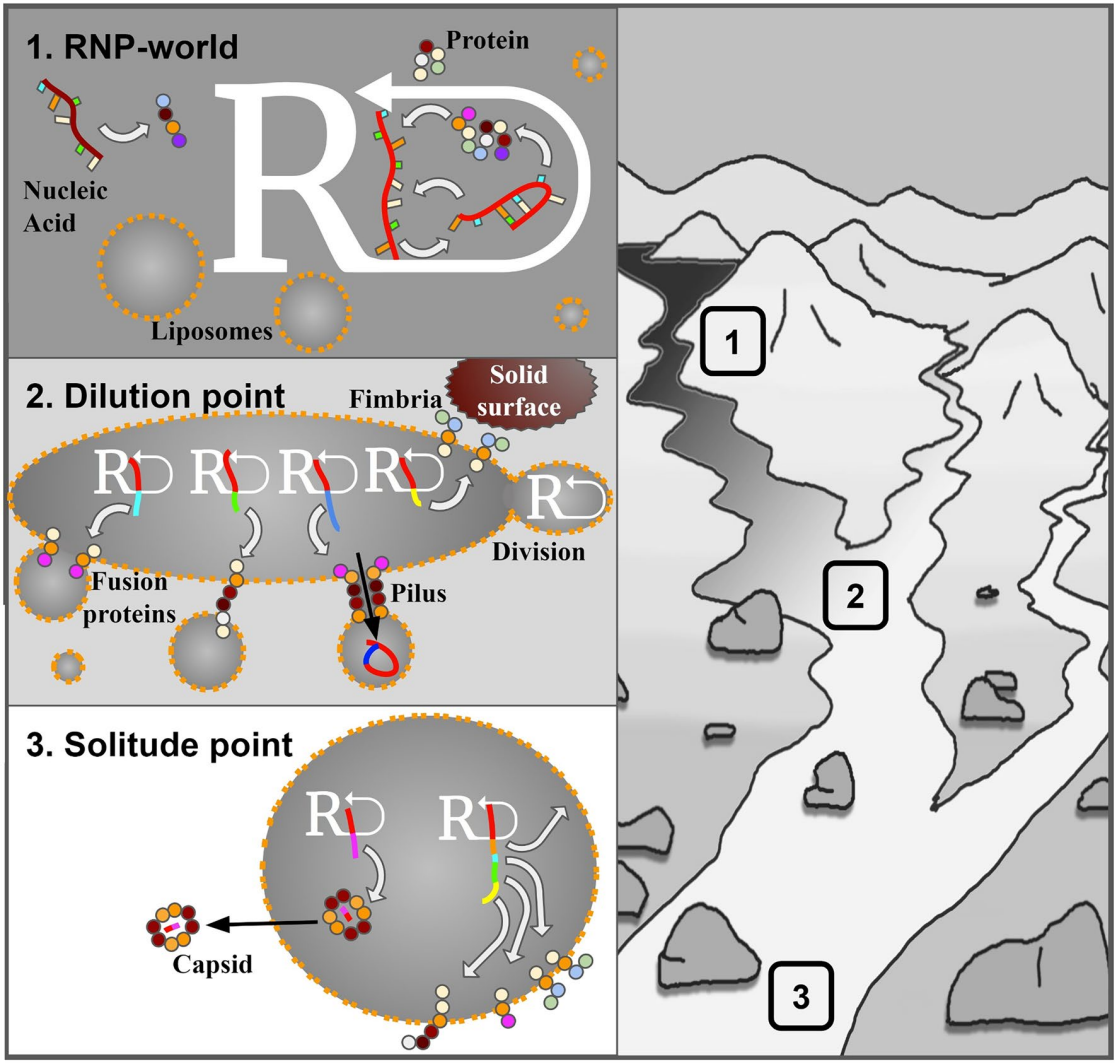
A graphical representation of the Bubble Theory, showing the potential landscape where the transition from first replicators to cells and viruses could have happened. The “warm little pond” **(1)** full of liposomes-forming amphiphiles (orange) and nutrients (activated building blocks) needed for the RNP world, is shown in dark. The gradient of nutrients diluted by the tributaries of “freshwater” is shown as a transition from dark to light color. Single-strain information-storing nucleic acid is represented as a string with free nucleobases. Functional ribonucleic acid is represented as a nucleic acid with a secondary structure. Proteins/peptides are shown as colorful beads. A cis-acting nucleic acid that is able to fulfill an autocatalytic cycle (molecular biology dogma) in which its sequence is being replicated is shown in red. A self-maintained replicator/replicon is designated as a letter R with an arrow pointing back at it. Nucleic acids not involved in this process are colored in non-red colors. Dilution of the original nutrient solution leads to the formation of a dilution point **(2)** where replication of replicons is no longer possible. Only within the bubbles formed back in the source, replication can take place. Replicons must develop adaptations like fusion proteins for merging with other bubbles in order to provide a fresh portion of nutrients; pili to transfer their genetic information to other vesicles; and fimbriae needed for anchoring to the solid surfaces, preventing them from being washed away from the nutrition supply zone. Bubbles that grow large, become insatiable and divide. Bubbles washed out beyond the solitude point **(3)** have to gain new adaptations to survive. They must start to maintain their own physical boundary by producing lipid membranes and finding a new way to obtain nutrients. Their genotype becomes enriched with sequences coding for metabolic pathways providing needed chemicals. They become fully fledged cells. Other replicons have to send a copy of themselves in the form of a spore to change the host bubble. As the possibility of meeting the other bubble is low beyond the solitude point, the development of economic (minimalistic) capsid seems the most profitable. They become viruses

## Strategies

The cell strategy appears relatively straightforward, as the merging and division of liposomes have been observed to occur spontaneously (Hanczyc et al. 2003; Walde et al. 2010), potentially marking the beginning of nutrition and proliferation. However, evolving into a future cell was associated with a significant shift in survival mechanisms. Within bubbles, the strategy of being the fastest replicator becomes less profitable due to the new external environment and the emergence of new evolutionary pressures. The fastest replicators would deplete resources within the bubble and perish. Therefore, replicators adopting the cell strategy must have developed new tools that could serve desired purposes in the new external environment. Here, the rapid rate of mutation in the RNA world could have initially provided a source of genetic diversity and preadaptations, driving the development of new features within the bubble’s ecosystem and accumulating genetic information needed to survive beyond the dilution point.

Preadaptations, such as the production of transmembrane proteins (Fig. 1.2), could significantly increase the probability of bubble merger by reducing the fusion energy between two lipid bilayers (fusogenic transmembrane proteins). The emergence of such proteins would be advantageous and promoted by natural selection. The fusion process would be non-specific, leading to merged vesicles containing nutrition alone, replicons from Eden, or replicons with evolutionary adaptations to feed on other bubbles. This process would also facilitate the horizontal mixing of genetic information, which could be a beneficial process, reducing the time required to evolve a viable cell (Sinai et al. 2018).

Another evolutionary pressure in this environment would be the ability to remain within the fresh liposomes supply zone extending down the stream from the dilution point. As nutrients became increasingly diluted further away from the source, bubbles also became scarce, reaching a point where the chance of two bubbles meeting was close to zero (the solitude point) (Fig. 1.3). Therefore, the bubble would benefit from a preadaptation that allowed it to stay within the Goldilocks zone. A set of transmembrane proteins would also prove useful for this reason, as they could act as fimbriae, anchoring a bubble to rocks and boulders along the way, effectively transforming it into a predator awaiting prey.

The virus strategy was an adaptation of replicons rather than bubbles. Parasites, as direct descendants of the RNA/RNP world, remained with the fast replication tactics. However, in the bubble environment, they also had to develop new, sophisticated survival methods (as shown in Fig. 1.2). The simplest approach appears to be a byproduct of the previously mentioned cell strategy. As the proto-cell grew due to its fusogenic transmembrane proteins (Payne 2017), it became unstable and produced daughter bubbles through a spontaneous budding-like process. Natural selection would have favored adaptations that allowed the production of smaller information-containing buds, which could inoculate other bubbles at a lower energetic cost. Although there is only one known family of enveloped, capsid-lacking viruses—Plasmaviridae (Krupovic, 2018) that might be a result of secondary loss of this feature, there are also liposomes and exosomes that carry nucleic acids as cargo (Hessvik and Llorente 2018; Xie et al. 2022), indicating such a possibility. The parallel concept involves the use of transmembrane proteins as pilus-like structures, inoculating neighboring proto-cells with genetic information through the cooperation between proteins and nucleic acids, as assumed in the RNP world (Cech 2009).

A capsid could have emerged as a stabilizing protein (Forterre and Prangishvili 2009; Krupovic et al. 2019), capable of preserving a copy of the replicon, thus maintaining its information and integrity for longer than a naked molecule could. If such complexes could withstand a shortage of nutrients within the bubble until the time of new fusion, this solution would provide an evolutionary advantage to the replicon hidden within such a structure. However, if we assume that the genome of such an entity was similar to the smallest genome found in free-living bacteria (approximately 1.3 Mb with 1100 genes) (Giovannoni et al. 2005), the capsid required to store the entire information would have to be gigantic, at least the size of Mimivirus, which is 0.7 µm in diameter and can accommodate a 1.2-Mb genome (Claverie et al. 2009). Based on this estimation, it is plausible that the infectious role of the capsid evolved at a later stage when viruses became smaller. Thus, budding and pilus-like structures were likely the initial solutions to the problem of transmission.

Capsid- or bud-using viruses and certain pilus-using entities, now known as mobile genetic elements (MGEs) (Krupovic et al. 2019), are considered related entities with fundamentally similar strategies based on maintaining the transmission process and self-recognizing one's “body” as a specific replicon, as opposed to an enclosed physical space known as a cell. Regardless of the virus strategy employed, the evolutionary advantage of keeping genetic information on a single piece of nucleic acid would be favorable (Lawrence 1999; Lawrence and Roth 1996). In the case of liposomes containing only nutrients, a complete genome would need to be inserted (possibly with some enzymes/ribozymes) to kick-start the molecular machinery. Proto-cells, on the other hand, did not face such strong evolutionary pressure and could maintain their information in fragments—chromosomes.

As viruses were dependent on bubbles, an arms race must have started between parasites and hosts. Future cells had to develop defensive mechanisms to protect themselves against parasitic replicons that could deplete their resources and threaten the bubble’s survival. The development of RNA caps and exonucleases could have been among the mechanisms serving this purpose, as they would have protected the native nucleic acid and hydrolyzed invasive genetic material. Additionally, the transition from RNA to DNA, despite its association with viral enzymes, might have decreased the stability of RNA-based parasitic replicons. Alternatively, it could have been one of the parasitic strategies subsequently adopted by the proto-cells.

Regardless of its origin and purpose, the RNA to DNA shift must have taken place within the timeframe of the events described here. This transition, however, would be an orthogonal event not significantly affecting the hypothesis described here, as the entire scenario can be accommodated within both translational or transcription–translational paradigms. The development of DNA, however, regardless of when it occurred, was crucial for the further evolution of the bubble into a cell, as its stability allowed genetic information to accumulate.

Summarizing, within the supply zone, the encapsulation of replicons within bubbles exerted an evolutionary pressure for cooperation, driven by the interdependence of the bubble’s survival and that of its contents. This process led to the development of advantageous traits, such as the production of transmembrane proteins capable of anchoring the bubble or merging it with other liposomes. These adaptations allowed the bubble to stay within the nutrients supply zone against the water flow and maximize the merging/feeding opportunities. As replicons had a common interest in keeping their bubble equipped in all the newest survival tools, the fastest replicon selection paradigm disappeared in favor of less aggressive maintenance of the information. This was the beginning of the cell strategy. Alternatively, replicons for survivor could migrate between bubbles. For this reason, the transmission system must have been developed, most likely involving pilus-like transmembrane proteins or buds. This was the beginning of the virus’ way of life.

## Individuality and distinctness

It is important to note that at this stage, the strategies described as viruses or cells are not definitively classified into specific branches of entities, as virus budding and cell division appear to be related processes that could have been the same thing. Additionally, pili brought benefits to both strategies, as they are known to bring bacteria closer together and facilitate the transfer of nucleic acids/proteins (Grahn et al. 2000; Llosa et al. 2002). It seems that within a single bubble, there could have been a combination of cell-like and virus-like strategies, as depicted in Fig. 1.2. This is because, at this point, the individual entity was not the entire bubble itself, but rather the replicons residing within it. Bubbles were not considered part of the body in the way cell membranes are today; they simply determined the environment for the replicons. There could have been multiple replicons populating a single bubble, competing for resources. Thus, bubbles were consortia, functioning as ecosystems with the common goals of remaining within the supply zone and hunting new bubbles, with both individual and group natural selection influencing them (Szathmáry and Demeter 1987). However, they were not yet distinct organisms.

An individual replicon could have consisted of a nucleic acid molecule storing the necessary information for self-maintenance, such as replication and metabolism (housekeeping genes), along with a fragment responsible for additional features. Different proteins could provide the bubble with various properties and predetermine the replicon’s future evolutionary pathway. For example, if the additional feature was a pilus, the replicon would adopt a parasitic strategy as it could (with additional adaptations related to specificity) recognize its own sequence and move to another bubble, gaining time for replication before facing competition. If the protein promoted budding, it could facilitate the encapsulation of the replicon with a small portion of the proto-cytoplasm, serving the purpose of infecting other bubbles. However, replicons that coded for transmembrane proteins functioning as fibrils or promoting fusion were destined to become cells.

To become fully fledged individuals, both proto-cells and proto-viruses had to define their boundaries and develop a form of self-awareness in a non-cognitive manner. They achieved this through self-maintenance of their identity (Piast 2019).

Proto-cells became more efficient in hunting bubbles; however, they did not become distinct entities until they started maintaining the lipid bilayer that separated them from the environment. The solitude point likely played a crucial role in this metamorphosis. Bubbles deprived of a lipid source had to begin producing their own amphiphiles. This step defined the entity as self-maintaining its open system, identifying with it, and distinguishing itself from the environment (Fig. 1.3). The dawn of the first cells and life as we know it, has begun. Furthermore, the environment imposed a novel challenge for bubbles to develop strategies for acquiring essential building blocks required for their sustenance and emancipation from Eden’s production. Alternative nutrients sources were utilized, and alternative production pathways were deployed to adapt to the new surroundings. The development of a proton gradient across the membrane facilitated energy acquisition (Lane and Martin 2012), paving the way for the early versions of the photosynthesis, likely relying on reducing the ferredoxin (Martin et al. 2018).

Proto-viruses, on the other hand, found it evolutionarily advantageous to lose genes that coded much of the molecular machinery. With the increase in the presence of bubbles in the environment, duplicated housekeeping information became redundant. Instead, they evolved specific transmission systems recognizing viral nucleic acids and facilitating their spread. Hence, the core of viral genetic information primarily comprises genes governing self-maintenance (self-recognition, self-replication) and transmission (capsid structure, packaging, cell lysis, new host recognition) representing the essential minimum for parasitic existence (Chaitanya 2019). The remaining information, including protein production, is provided by the host.

Within the supply zone, where a population of bubbles is already numerous, the strategy of spreading from one host to another, increasing the number of the parasite copies in the population, regained profitability. If the infection was mild enough not to disrupt the bubbles functionality, parasitic replicons could spread across the supply zone via physical contact through pili and buds. However, this system proved infective closer to the solitude point, as proto-cells might have been too distant for direct contact. Parasitic replicons become confined to vertical transition that exposed them to the evolutionary pressure, tempering their viral properties in favor of cooperation. This is where production of small, energetically cheap particles—capsids have become the most advantageous strategy from the virus point of view. It significantly increased the likelihood of finding a new host and allowed viruses to entirely separate themselves from the bubbles in the form of infectious spores—virions (Fig. 1.3). This step further catalyzed the reduction of genome size, allowed lysogeny as a strategy aiming to maximize the hosts resources and led to the formation of viruses as we know them today.

## Closing remarks

This paper has explored how early replicators began to establish their distinctiveness as cells or parasites. It does not delve into topics such as monomer production, energy acquisition, faithful replication, or metabolic pathways, as it assumes these traits were already present in replicators or provided by the environment. The sustainability of the RNP world, which serves as a functional imperative for this work, is taken as given.

The Bubble Theory scenario can be considered as a subsequent stage following the garbage bag model proposed by Freeman Dyson (1999) and the lipid world concept (Segré et al. 2001) wherein external selective pressure dictates the trajectory of changes toward specific evolutionary adaptations. A natural consequence of these changes seems to be development of cell-like structures or virus-like strategies.

The essence of this proposal resonates with the idea that early forms of life continuously exchanged genetic information as a collective rather than existed as individual organisms (Vetsigian et al. 2006; Doolittle 1999). It describes bubbles as environments that provided an evolutionary pressure for enclosed within them protein-producing replicons. This selection was promoting the cooperation between replicons by establishing a preferable set of abilities, a bubble should have to endure in the external environment. The conditions allowing this transition were provided by the dilution of nutrients caused by the tributary of the prebiotic river flowing from the Darwinian “warm little pond.” The emergence of a cooperation between replicons would be a consequence of The Game Theory principles described in “The Evolution of Cooperation” by Axelrod and Hamilton (1980). The selfish replicons, on the other hand, adopted a parasitic strategy and gave rise to virus-like entities. The adoption of either the cell or virus strategy was advantageous for ancestral replicons navigating an environment where nutrients (the original primordial soup) were confined within liposomes. The consequence of further specialization in these strategies was the emergence of viral and cellular lineages by self-definition of their boundaries (Buss 2014) and evolutionary transmission in individuality (West et al. 2015).

The Bubble Theory offers insight into the origins of cells and viruses, presenting a logical progression of adaptations driven by natural selection. It is important to note that this scenario, situated at the very beginning of life, does not exclude alternative hypotheses regarding the origin of viruses but can be viewed as a complementary approach.

In conclusion, this paper sheds light on the emergence of distinct replicator entities and their strategies in the early stages of life as they could have emerged on the rocky worlds with liquid water like the Earth, and perhaps in the case of different chemistry life, also for rocky worlds with other liquids present on their surfaces like in the case of Titan. Further research and investigation are required to fully unravel the complexities of this topic.
